# Novel Approach to Simulate Sleep Apnea Patients for Evaluating Positive Pressure Therapy Devices

**DOI:** 10.1371/journal.pone.0151530

**Published:** 2016-03-15

**Authors:** Valentina Isetta, Josep M. Montserrat, Raquel Santano, Alison J. Wimms, Dinesh Ramanan, Holger Woehrle, Daniel Navajas, Ramon Farré

**Affiliations:** 1 Unitat de Biofísica i Bioenginyeria, Facultat de Medicina, Universitat de Barcelona, Barcelona, Spain; 2 CIBERES, Madrid, Spain; 3 Sleep Laboratory, Pneumology Department, Hospital Clinic, Barcelona, Spain; 4 Institut d'Investigacions Biomèdiques August Pi i Sunyer, IDIBAPS, Barcelona, Spain; 5 ResMed Science Centre, Munich, Germany; 6 Institute for Bioengineering of Catalonia, IBEC, Barcelona, Spain; Georgia State University, UNITED STATES

## Abstract

Bench testing is a useful method to characterize the response of different automatic positive airway pressure (APAP) devices under well-controlled conditions. However, previous models did not consider the diversity of obstructive sleep apnea (OSA) patients’ characteristics and phenotypes. The objective of this proof-of-concept study was to design a new bench test for realistically simulating an OSA patient’s night, and to implement a one-night example of a typical female phenotype for comparing responses to several currently-available APAP devices. We developed a novel approach aimed at replicating a typical night of sleep which includes different disturbed breathing events, disease severities, sleep/wake phases, body postures and respiratory artefacts. The simulated female OSA patient example that we implemented included periods of wake, light sleep and deep sleep with positional changes and was connected to ten different APAP devices. Flow and pressure readings were recorded; each device was tested twice. The new approach for simulating female OSA patients effectively combined a wide variety of disturbed breathing patterns to mimic the response of a predefined patient type. There were marked differences in response between devices; only three were able to overcome flow limitation to normalize breathing, and only five devices were associated with a residual apnea-hypopnea index of <5/h. In conclusion, bench tests can be designed to simulate specific patient characteristics, and typical stages of sleep, body position, and wake. Each APAP device behaved differently when exposed to this controlled model of a female OSA patient, and should lead to further understanding of OSA treatment.

## Introduction

Obstructive sleep apnea (OSA) is a prevalent breathing disorder and is considered a major public health issue, affecting 5–15% of the general population and increasing with both body mass index and age (up to at least 60–65 years) [1,2]. OSA is characterized by repetitive narrowing and closure of the upper airway during sleep [3] that results in brain arousal, intermittent hypoxia, negative intrathoracic pressure swings, and increased sympathetic activity. OSA is associated with a reduction in quality of life, daytime sleepiness, traffic accidents, neurocognitive impairment, metabolic, cardiovascular disease [4] and malignancies [5].

The treatment of choice for OSA is the application of continuous positive airway pressure (CPAP) to the patient’s nose or mouth through a mask during sleep at home. This pressure in the mask is transmitted to the pharyngeal area, splinting the collapsible upper airway walls thereby avoiding obstruction. Auto-adjusting positive airway pressure (APAP) devices, which are increasingly being used, are driven by algorithms that measure abnormal sleep breathing events, analyze the patient’s breathing pattern and eventually increase the delivered pressure in response to airway obstruction, or decrease pressure when breathing is stable to increase patient comfort [6–11]. In theory, APAP devices should be ideal for treating a range of patients with different characteristics, and for effectively treating OSA despite within-night and night-to-night variations in the upper airway collapsibility experienced by each individual patient [12–16]. However, commercially available APAP devices contain undisclosed proprietary algorithms, and therefore the way that they measure and respond to specific breathing patterns varies [17]. In addition, some APAP manufacturers are introducing new algorithms based on specific patient characteristics. This move towards personalized medicine in the treatment of OSA means greater choice for patients and more variability in APAP algorithms. Therefore, understanding how each device responds to different OSA patterns requires comparative studies using well defined references.

Bench testing is a useful method to characterize the response of different APAP algorithms under well-controlled conditions, thus avoiding the biological variability inherent in clinical trials. However, previously used bench test models have been based on subjecting the APAP device under test to a repetitive string of disturbed breathing patterns, without providing a sufficiently wide spectrum of events. These limitations mean that variety in patient characteristics and phenotypes, or the changes that occur during different sleep stages and body positions over the course of a night’s sleep, cannot be taken into consideration. This is particularly relevant given that different subpopulations of OSA patients (e.g. children, men, women, the elderly) exhibit specific traits in their sleep-related breathing disorders [18].

Therefore, the aims of this proof-of-concept study were: 1) to design a new complex and versatile bench test approach for realistically simulating respiratory events throughout the course of the night in an OSA patient, mimicking breathing disturbances across different phenotypes, and 2) to implement a full night example of a female OSA phenotype and use this to compare the responses of several currently-available APAP devices.

## Materials and Methods

The hardware of our new model was based on a previously described bench test [19]. This fully computer-driven model comprises a servo-controlled pump able to deliver a flow that replicates any breathing waveform stored in the computer. An obstruction valve allows the simulation of controlled obstructive events by imposing mechanical impedances previously recorded in patients with OSA. Two other valves can mimic leaks and mouth breathing, and a loudspeaker-in-box system can superimpose simulated snoring onto the breathing flow. The test bench is equipped with two sensors, one to measure pressure at the simulated patient entrance and one to measure the actual flow generated by the patient simulator. A calibrated leak based on a 4-mm internal diameter (ID) orifice [20] mimics the mask leak (exhalation port) in nasal masks. In previous studies, this system was fed by a collection of disturbed breathing events, such as obstructive and central apneas, hypopneas, flow limitation, mask leaks and mouth expiration [19,21].

To design the new OSA simulator model we developed a novel approach aimed at realistically replicating a typical night of sleep for a female patient. With this aim, we considerably expanded our library of disturbed breathing patterns anonymously extracted from polysomnography recordings obtained from real OSA patients and we incorporated several new adjustable features into the simulator. Specifically, the new patient model can be set to react to the pressure delivered by the APAP device (PAP-responsive mode) or to reproduce a fixed scenario of disturbed breathing events (Steady mode), depending on the device characteristics being tested. Moreover, the severity of the simulated OSA profile is now fully modifiable by changing the frequency and duration of each breathing event. Various artefacts were introduced into the event spectrum, such as changes in tidal volume and breath rate, to replicate typical events during wake such as irregular breathing, swallowing, moving and talking. By combining these new features, we aimed to create a new OSA model concept model that can realistically replicate a whole night of sleep, including phases of wake, rapid eye movement (REM) and non-REM sleep, and change in body position, each one designed to mimic different characteristics in terms of upper airway collapsibility.

**Fig 1:**
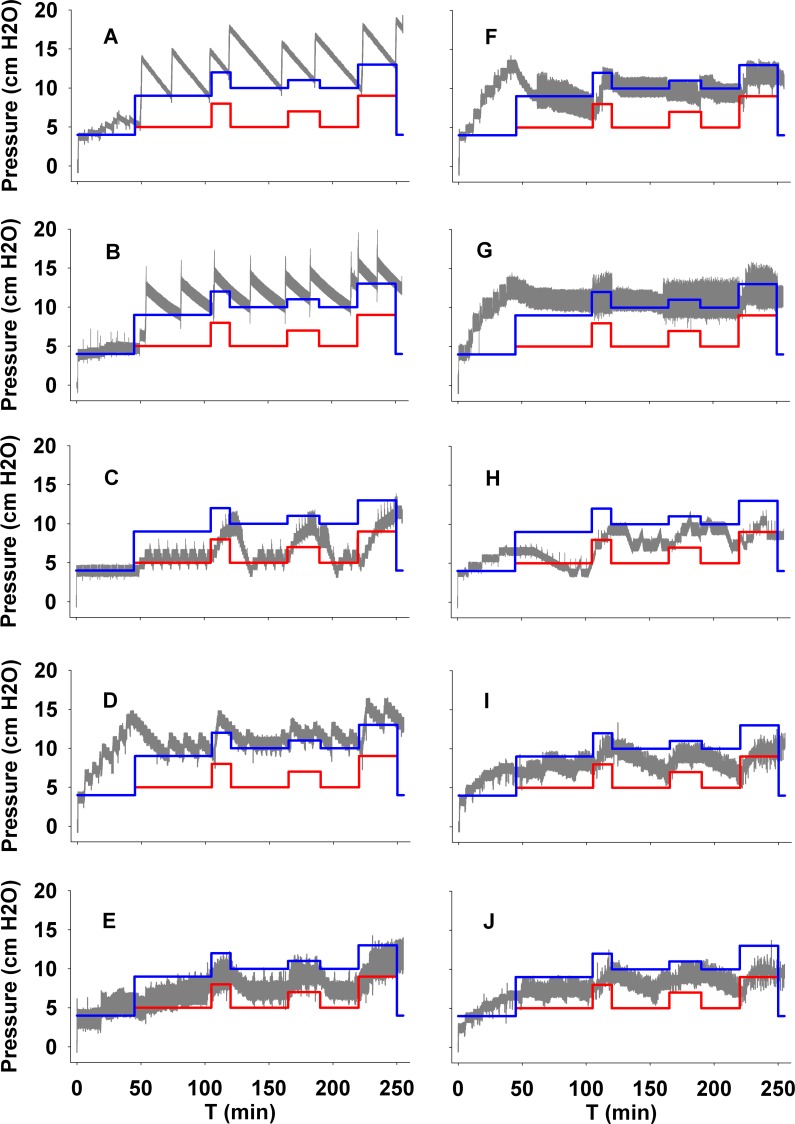
Pressure trends over a full simulated night (grey line) for all APAP devices tested. A device that delivered pressures above the blue line achieves full breathing normalization, while if it delivered pressures just above the red line only obstructive apneas were overcome.

For this study specifically, as an example of an entire night of sleep-disordered breathing (SDB), the bench test model was set to simulate the disturbed patterns of a female OSA patient with the following characteristics: long sleep latency (45 min), low positive airway pressures (PAPs) required to overcome obstructive events, high proportion of flow limitation events versus apneas, higher apnea-hypopnea index (AHI) during REM sleep, and only minor positional effects on upper airway collapsibility. The features and structure of this female-specific OSA patient simulation are detailed in Table 1. The breathing pattern of the simulated patient depended on the PAP applied by the device under test, with a total duration of 4 hours and 15 minutes. APAP pressure values required to normalize breathing during each stage of the simulation are shown in Fig 1. The simulated night consisted of programming the different stages described in Table 1, starting with 45 minutes of simulated awake stage (sleep onset) followed by a succession of different sleep stages with the features detailed in Table 1 (e.g. breathing frequency, number and types of respiratory events) and a final awake short period. In this way we were able to model a patient exhibiting different sleep breathing characteristics throughout consecutive sleep stages.

**Table 1:** Description of the patient simulation implemented in the bench test model.

Stage	Duration	AHI	Features
Sleep onset	45 min	-	
			16 breaths/min
			V_T_ 500 mL
			Random insertion of changes in breathing rate and V_T_, and swallowing
Non-REM cycle 1	60 min	15/h	
			Body position: side
			Apneas (0–5 cmH_2_O): event length 12 sec
			Hypopneas (5–7 cmH_2_O): event length 16 sec
			Flow limitation (7–9 cmH_2_O)
			Normal breathing (>9 cmH_2_O)
REM cycle 1	15 min	30/h	
			Apneas (0–8 cmH_2_O): event length 18 sec
			Hypopneas (8–10 cmH_2_O): event length 16 sec
			Flow limitation (10–12 cmH_2_O)
			Normal breathing (>12 cmH_2_O)
Non-REM cycle 2	45 min	15/h	
			Body position: side
			Apneas (0–5 cmH_2_O): event length 12 sec
			Hypopneas (5–7 cmH_2_O): event length 16 sec
			Flow limitation (7–10 cmH_2_O)
			Normal breathing (>10 cmH_2_O)
REM cycle 2	25 min	30/h	
			Apneas (0–7 cmH_2_O): event length 18 sec
			Hypopneas (7–9 cmH_2_O): event length 16 sec
			Flow limitation (9–11 cmH_2_O)
			Normal breathing (>11 cmH_2_O)
Non-REM cycle 3	30 min	15/h	
			Apneas (0–5 cmH_2_O): event length 18 sec
			Hypopneas (5–7 cmH_2_O): event length 16 sec
			Flow limitation (7–10 cmH_2_O)
			Normal breathing (>10 cmH_2_O)
REM cycle 3	30 min	30/h	
			Body position: supine
			Apneas (0–9 cmH_2_O): event length 18 sec
			Hypopneas (9–11 cmH_2_O): event length 16 sec
			Flow limitation (11–13 cmH_2_O)
			Normal breathing (>13 cmH_2_O)
Awake	5 min	-	Normal breathing

AHI: apnea-hypopnea index; REM: rapid eye movement; V_T_: tidal volume.

Ten different commercially available APAP devices were tested using the new bench test model and the female-specific simulation described above: AirSense 10 (A) and AirSense 10 AutoSet for Her (B) by ResMed; Dreamstar by Sefam (C); Icon by Fisher & Paykel (D); Resmart by BMC (E); Somnobalance (F) and Prisma 20A (G) by Weinmann; System One by Respironics (H); iCH (I) and XT-Auto by Apex (J). Each APAP device was connected with its own tube to the bench model. Default APAP settings were used (minimum pressure 4 cmH_2_O, maximum pressure 20 cmH_2_O). Each device was tested twice and the results averaged to obtain the final values.

## Results

The new OSA patient simulator could effectively combine a great variety of SDB elements to mimic the response of the predefined patient type. The responses of the assessed APAP devices to the new female-specific bench test model are summarized in Table 2. There was considerable variation among devices, particularly with respect to the mean and maximum nasal pressures applied, and the ability to overcome obstructive events and flow limitation, The residual AHI was calculated as the number of residual obstructive events per hour and the residual flow limitation was measured as the portion of the test in minutes (excluding the initial 45-minute wake period) that the simulated patient remained on flow limitation.

**Table 2:** Reponses of automatic CPAP devices to a specific simulated OSA patient.

Device	P_max_, cmH_2_O	P_mean_, cmH_2_O	Residual AHI, /h	Overcome obstructive events?	Overcome flow limitation?	Residual flow limitation, min (% sleep time)
**A**	18.65	13.25	0.7	Yes	Yes	4 (2%)
**B**	15.4	11.8	0.7	Yes	Yes	4 (2%)
**C**	11.4	6.75	16.5	No	No	24 (12%)
**D**	15.3	11.3	0.6	Yes	Yes	24.5 (12%)
**E**	11.35	7.7	11.9	No	No	81 (40%)
**F**	12.6	9.5	2.4	Yes	No	167 (81%)
**G**	12.1	10.05	1.6	Yes	No	122 (60%)
**H**	12.45	7.75	10	No	No	76 (37%)
**I**	10.6	8.3	6.5	Yes	No	142 (69%)
**J**	10.1	8.2	8.5	No	No	132.5 (65%)

AHI: apnea-hypopnea index; P_max_: maximum positive airway pressure applied; P_mean_: mean positive airway pressure; A: AirSense 10 by ResMed; B: AirSense 10 AutoSet for Her by ResMed; C: Dreamstar by Sefam; D: Icon by Fisher & Paykel; E: Resmart by BMC; F: Somnobalance by Weinmann; G: Prisma 20A by Weinmann; H: System One by Respironics; I: iCH by Apex; J: XT-Auto by Apex.

Breathing normalization with a residual AHI <5/h was only achieved with devices A, B and D; devices E, H, I and J were associated with more than five residual events per hour. Pressure changes of each device throughout the whole test are displayed in Fig 1.

**Fig 2:**
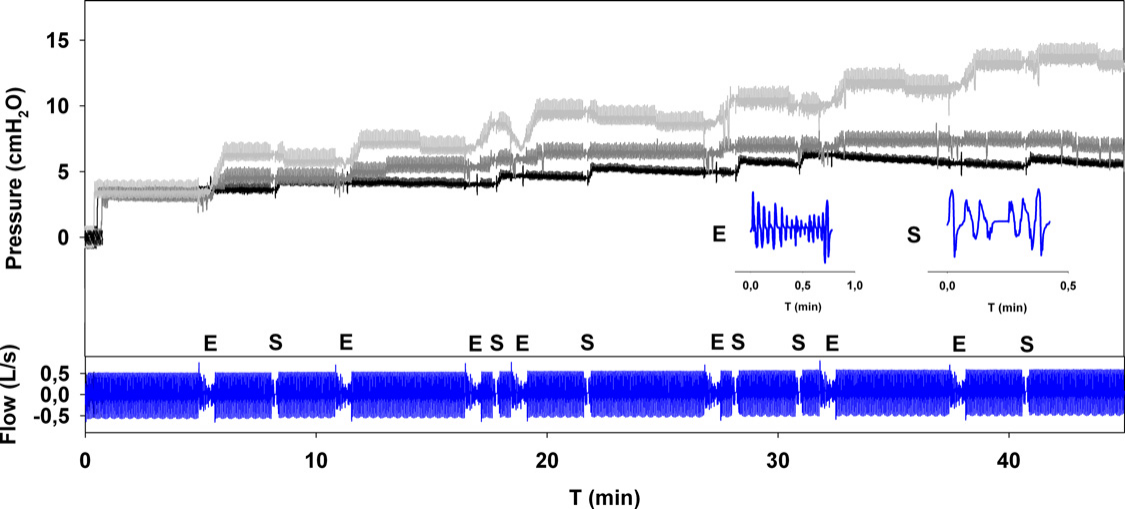
Pressure trends for three different APAP devices tested during the initial 45-minute simulated wake period. Device A (black line) showed a mild pressure increase (< 2 cmH_2_O), device I (dark grey line) showed a moderate pressure increase (2.5–3 cmH_2_O), while device D (light grey line) showed a high pressure increase (>7 cmH_2_O) in response to the breathing pattern simulating 45 minutes of wake period (blue line). E: erratic breathing; S: swallowing.

Considering the 45-minute wake period, there was significant variation in the behaviour of the different devices. Table 3 shows the pressure values reached by each tested device at the end of the simulated wake period. Device C did not increase the pressure during wake periods. Three devices (A, B and E) displayed only mild pressure increases (<2 cmH_2_O). Moderate pressure increases (2.5–3 cmH_2_O) were displayed by three devices (H, I and J), and significant pressure increases (>7 cmH_2_O) were seen from three devices (D, F and G). Three examples of different responses during the simulated wake period are presented in Fig 2, together with the flow signal generated by the simulator during the initial awake phase, which consisted of normal breathing with some events inserted simulating flow alterations due to irregular breathing (E) and swallowing (S). Devices A, B and D contain algorithms aimed at automatically detecting sleep onset (for A, B AutoRamp mode and for D SenseAwake mode). Devices A and B showed similar pressure increases with AutoRamp mode turned off, while device D responded with higher pressure increases when the SenseAwake mode turned off.

**Table 3:** Pressure values reached by each device after 45 minutes of simulated wake.

Device	APAP pressure after 45 min of simulated wake (cmH_2_O)
**A**	5.4 (5.8 with AutoRamp OFF)
**B**	4.8 (5.2 with AutoRamp OFF)
**C**	4.0
**D**	11.2 (14.5 with SenseAwake OFF)
**E**	4.6
**F**	11.8
**G**	11.7
**H**	6.5
**I**	6.8
**J**	6.9

A: AirSense 10 by ResMed; B: AirSense 10 AutoSet for Her by ResMed; C: Dreamstar by Sefam; D: Icon by Fisher & Paykel; E: Resmart by BMC; F: Somnobalance by Weinmann; G: Prisma 20A by Weinmann; H: System One by Respironics; I: iCH by Apex; J: XT-Auto by Apex.

To assess whether the observed variations in pressure during wake had an influence on the results of testing, a subset of devices that showed a moderate to significant pressure increase during sleep onset (D, G, H and I) were retested without the wake phase of the test. In this additional analysis (Table 4), the responses of the tested devices were relatively similar to the ones in the previous tests that included the 45-minute sleep onset phase. The largest change was seen in device D, where the residual AHI increased from 0.6 to 6 events per hour.

**Table 4:** Results of device re-testing without the sleep onset period.

Device	P_max_, cmH_2_O	P_mean_, cmH_2_O	Residual AHI, /h	Overcome events	Overcome flow limitation	Residual flow limitation, min (% sleep time)
**D**	14.6	8.95	6	Yes	Yes	9 (4%)
**G**	11.65	9.25	2.6	Yes	No	164 (80%)
**H**	11.45	7.35	6.6	No	No	70 (34%)
**I**	11.3	7.9	9.6	Yes	No	107.5 (52%)

AHI: apnea-hypopnea index; NA: not available; P_max_: maximum positive airway pressure applied; P_mean_: mean positive airway pressure; D: Icon by Fisher & Paykel; G: Prisma 20A by Weinmann; H: System One by Respironics; I: iCH by Apex.

## Discussion

We successfully developed and carried out a proof-of-concept test of a novel optimized bench model easily adaptable to simulate different SDB patterns found in OSA, including periods of wake, periods representing different sleep stages and phases of more or less severe SDB events. This tool can be useful to objectively evaluate bench test performance of different APAP devices with realistic breathing patterns covering a wide range of patient phenotypes. In its “Steady mode”, the simulator could also assess the capacity of APAP, as well as CPAP, devices to estimate treatment duration and detect residual respiratory events of a fixed predefined disturbed breathing scenario.

The presentation and severity of OSA varies greatly depending on patient characteristics such as gender, age, body mass index, and craniofacial structure [18,22]. Specific patient subgroups have been gaining a lot of attention recently because of their clinical relevance. At one end of the age spectrum, elderly patients tend to present with severe OSA and snoring becomes less common. In addition, the frequency of central events increases, although obstructive events still predominate [23]. In contrast, children with OSA have frequent snoring, restless sleep, mouth breathing, apneas, gasping, and laboured or paradoxical breathing [24]. With the growing trend towards personalized therapy, specific patient breathing patterns will be increasingly studied as manufacturers work to design the most optimal treatment for each phenotype.

One good example of this is OSA in females versus males. It is well-known that the polysomnographic features of female OSA are different from those of male OSA. Overall, women have less severe OSA with, on average, a lower AHI [25] and shorter apneas [26]. Women also have more episodes of upper airway events during REM sleep [25]. Body position is far less important for the severity of OSA in women, while OSA severity in men is based more on position than sleep state [25]. Furthermore, women may take longer to fall asleep, but have fewer awakenings during sleep [27]. Regardless of the patient’s gender, there is also significant night-to-night variation in OSA, based on factors such as body posture, sleep stages, and previous drug or alcohol intake [28]. Besides OSA pathophysiology, gender influences also patients’ PAP requirements [29], as generally female patients require lower pressures. Such considerable variability between phenotypes highlights the relevance of the simulation approach taken in this study. In our optimized bench test we implemented a dynamic pattern (“PAP-responsive”) simulating a female patient phenotype (although an individual male patient may also present with this OSA pattern), which included long periods of flow limitation, low AHI, and short, low-severity obstructive events. Only three of the APAP devices tested were able to achieve full breathing normalization by overcoming all types of disturbed events including flow limitation. Considering the potential for increased flow limitation in female patients, which may lead to breathing disturbances, the effectiveness of treatment in patients presenting with a high component of flow limitation should be carefully examined.

Published data comparing different APAP algorithms is scarce, particularly for devices recently launched into the market. Pevernagie *et al* examined two APAP devices and found that the residual apnea-hypopnea index (AHI) was lower during use of one device compared with the other (3.5±5.6/h vs 9.9±31.0/h), and that the amount of snoring during the night was significantly higher with one device [30]. A similar study by Nolan *et al* compared three commercially available devices. The authors found that mean pressure and patient compliance were significantly lower on one of the APAP devices [17]. Differences between algorithms combined with a lack of information regarding how different auto-adjusting devices work has led to the perception that auto-adjusting devices are a ‘black box’ which should be used with caution [31]. In this study, we also found considerable variation among devices in both the magnitude of response to obstructive events, the time taken to increase pressure during disrupted breathing, and device behaviour during the simulated wake period. With the exception of one device, which did not increase the pressure at all, most devices at least slightly increased pressure during simulated wakefulness. Some devices showed quite an intense pressure response during the wake period of the test, with one reaching almost 14 cmH_2_O and two reaching 12 cmH_2_O. Due to the potential impact this could have on patient comfort, pressure changes during wake periods should be assessed in clinical practice, particularly in patients who report difficulties falling asleep while using PAP therapy or issues with comfort at higher PAP pressures.

As stated above, our finding of considerable variability in the response of APAP devices when subjected to the same breathing pattern under well-controlled conditions is in agreement with previous reports [19,21,32]. These variations can be attributed to the individual algorithms within each APAP device. Each algorithm analyses flow and pressure to determine whether there is a breathing disturbance, and then initiates the most appropriate response to correct such a disturbance. For instance, it is interesting to note that, as we explained previously [21], the simulated hypopneas in our model were defined according to specific values of a flow-limitation pattern index initially introduced by Teschler et al [33]. Therefore, it could be possible that automatic CPAP devices set to detect hypopneas using this index, or something similar, could be more suitable for detecting our simulated events than other devices that use other metrics to define and detect hypopneas. Another reason for the observed different response in the automatic CPAP devices tested is that the optimal rate of pressure increase after detection of obstructive events has not been clinically defined. In fact, APAP devices are designed to normalize breathing at a rate which treats actual SDB, avoiding any response to false events, thereby unnecessarily modifying pressure. The results of this bench test have shown that, under well-controlled conditions, there are marked variations in response by different APAP devices, and that there may be high residual AHI or uncontrolled flow limitation in some female patients on some APAP devices. Therefore, all APAP devices should not be considered equal, and efficacy and patient comfort should be carefully examined following APAP initiation.

It must be noted that our results are restricted to the specific patterns of disturbed breathing used in this bench test to simulate a specific OSA patient. It is possible that the response of the tested devices would have been different from the ones reported here if SBD was simulated using different patterns or patient phenotypes. In addition, a limitation of this study is that one device of each type was used. Hence, a more complete assessment would require testing of a larger number of each type of device randomly obtained from those available in the market. Finally, it should be stressed that although bench testing is a useful way to investigate the behaviour of different devices, testing outcomes may vary in clinical practice due to the almost unlimited spectrum of events and phenotypes found in real life. Indeed, crucial factors such as changes in loop gain, and upper airway compliance and pharyngeal critical pressure are not considered in our model. Accordingly, bench testing should be considered as a preliminary assessment before clinical evaluation in patients.

In conclusion, this study showed that a dynamic bench model tailored to represent specific OSA patient phenotypes, incorporating a variety of disturbed breathing events within the same simulated night, including different degrees of severity along sleep stages, and a period of wakefulness, can be useful to characterize treatment responses of commercially-available APAP devices. This demonstrates that bench testing can be modified to better represent a “real” patient, and that APAP devices can show markedly different responses to the same simulated breathing patterns. Realistically mimicking OSA patients during bench testing is useful as a first step to aid in the understanding of actual APAP device responses observed in the clinical setting, and can be helpful in selecting the device that best meets the individual needs of each patient, thereby improving comfort and increasing adherence to therapy, which is essential for effective treatment and reducing the consequences of OSA [34].
